# Impact of liver transplantation on the quality of life of a cohort of high-risk recipients

**DOI:** 10.31744/einstein_journal/2025AO0565

**Published:** 2025-03-28

**Authors:** Heloisa Barboza Paglione, Daisa de Mesquita Escobosa, Beatriz Mesquita Pimenta, Bianca Della-Guardia, Amanda Pinter Carvalheiro da Silva Boteon, Yuri Longatto Boteon

**Affiliations:** 1 Transplant Centre Hospital Israelita Albert Einstein São Paulo SP Brazil Transplant Centre, Hospital Israelita Albert Einstein, São Paulo, SP, Brazil.; 2 Health Economics Department Hospital Israelita Albert Einstein São Paulo SP Brazil Health Economics Department, Hospital Israelita Albert Einstein, São Paulo, SP, Brazil.; 3 Faculdade Israelita de Ciências da Saúde Albert Einstein Hospital Israelita Albert Einstein São Paulo SP Brazil Faculdade Israelita de Ciências da Saúde Albert Einstein, Hospital Israelita Albert Einstein, São Paulo, SP, Brazil.

**Keywords:** Liver transplantation, Quality of life, Liver cirrhosis, Alcoholism, Waiting list, Health status, Surveys and questionnaires, Morbidity

## Abstract

**Objective:**

To assess the effects of liver transplantation on the health-related quality of life of patients with cirrhosis on the waiting list for transplantation.

**Methods:**

This retrospective study analyzed HRQOL using the EQ-5D questionnaire before (T0), and 3 (T3), 6 (T6), and 12 (T12) months after, transplantation. The questionnaire comprised five health status scores, which were also combined into a single utility score. Changes in each dimension and in the utility score were examined.

**Results:**

A total of 212 patients were included in the study, with a mean age of 54 (SD, 12) years, and a median Model for End-Stage Liver Disease score of 17 (Q1= 11; Q3= 24). The most frequent etiology was chronic alcoholism (26.9%). Improvements in EQ-5D utility scores were registered at all post-transplantation time points compared to the score at T0: an increase of 0.22 points (p<0.001) at T3, 0.27 (p<0.001) at T6, and 0.268 (p<0.001) at T12. Scores increased over time in all domains of the EQ-5D.

**Conclusion:**

In a large sample of high-risk transplant recipients, a significant improvement in HRQOL occurred three months after transplantation, and was sustained over the one-year follow-up period.

## In Brief

Liver transplantation significantly improves the health-related quality of life of patients with cirrhosis and high MELD scores. This study highlights rapid health-related quality of life gains after transplantation that were sustained during the one-year follow-up period, emphasizing the role of multidisciplinary care in achieving these outcomes.

## Highlights

■ Liver transplantation improves health-related quality of life in patients with cirrhosis and high MELD scores.

■ Significant health-related quality of life improvements occur within three months of transplantation.

■ Multidisciplinary care programs enhance post-iver transplantation physical and mental recovery.

■ Sustained health-related quality of life gains highlight the long-term benefits of liver transplantation.

## Graphical abstract

**Figure:**
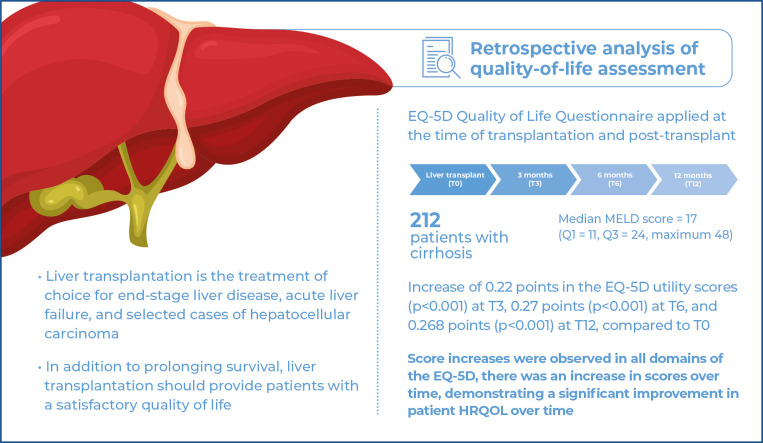

## INTRODUCTION

Liver transplantation (LT) is the treatment of option for end-stage liver disease, acute liver failure, and selected cases of hepatocellular carcinoma, among other emerging indications.^1^ It is performed with the aim of curing hepatic diseases and prolonging patient survival. These aims have been met owing to advances in surgical techniques, perioperative care, and the management of postoperative complications. Currently, the reported one-, three-, and five-year survival rates of patients undergoing LT are 86%, 77%, and 69%, respectively.^2^ However, in addition to prolonging survival, LT should provide patients with a satisfactory quality of life and enable them to partially or fully recover their ability to perform daily activities, including work.^3^

Health-related quality of life (HRQOL) encompasses social, mental, emotional, and physical domains in a multidimensional model.^4^ The systemic and metabolic changes caused by liver cirrhosis can negatively affect all of these domains.^5^ Furthermore, cirrhosis causes sarcopenia, malnutrition, and frailty, increases the risk of pre-transplant mortality, and delays post-transplantation physical function recovery.^6,7^ Studies have shown significant improvements in patient HRQOL following transplantation, owing to fewer restrictions on social activities and improved well-being.^8^However, the World Health Organization recommends that quality of life be considered in the context of the culture and value systems in which a person lives.^9^ Therefore, HRQOL is additionally affected by regional, economic, and cultural factors.

Few studies have investigated the impact of transplantation on the HRQOL of LT recipients in Latin America, although liver disease has been shown to significantly decrease their HRQOL.^8,10^ The findings of the present study may guide the proposals for improvements in patient care and changes in the clinical practices of the multidisciplinary teams.

## OBJECTIVE

This study aimed to examine the effects of liver transplantation on the health-related quality of life of patients with cirrhosis on the waiting list for transplantation.

## METHODS

### Study design, patient selection, and ethical considerations

This retrospective cohort study included patients who underwent LT between January 2016 and December 2020 as part of the Transplant Program of the *Hospital Israelita Albert Einstein* in São Paulo, Brazil. Data were obtained from a prospectively maintained retrospective database. The inclusion criteria were as follows: aged ≥18 years and electively listed for LT. The exclusion criteria were as follows: transplantation due to acute liver failure, transplantation of multiple organs required, and failure to complete the questionnaire. The study was reviewed and approved by the Research Ethics Committee of *Hospital Israelita Albert Einstein* (CAAE: 43746921.1.0000.0071; # 4.707.483).

### Quality of life instrument

The EQ-5D is a generic HRQOL questionnaire developed by the European Quality of Life Group,^11,12^ and has been validated following translation into Brazilian Portuguese.^13^ It comprises five health state description questions and a visual analog scale (VAS). The five health state description questions each analyze a separate dimension: mobility, self-care, usual activities, pain/discomfort, and anxiety/depression. Each dimension has three possible response levels: 0, no problems; 1, some problems; and 2, extreme problems. Therefore, 243 different health states are possible. The VAS allows respondents to rate their health on a numeric scale from 0 (“worst imaginable health state”) to 100 (“best imaginable health state”). The questionnaire results can be reported as five health status scores, or each health state can be ranked and transformed into a single score called the utility. The utility scores range from -0.59 to 1, where 1 stand for full health e 0 means death. Negative scores indicate health states worse than death. The details on the calculation of the score can be found in the original publications of the score.^11,12^

### Data collection

Data on pre-transplant demographic and clinical characteristics, such as cirrhosis a etiology, comorbidities, age, sex, Model for End-Stage Liver Disease (MELD) score, length of hospital stay, time on the waiting list, and transplantation details were collected. In addition, perioperative mortality, classified as death related to the procedure occurring within one year after transplantation, was determined. The EQ-5D questionnaire was administered prior to LT during the outpatient clinical assessment for inclusion in the transplant waiting list, and at 3, 6, 9, and 12 months postoperatively. All information was obtained from a retrospective institutional database prospectively maintained by the hospital health economics and transplant management teams, and was anonymized prior to delivery to the researchers.

### Statistical analysis

Quantitative variables are expressed as the mean and standard deviation (SD) or minimum and maximum or as median and quartiles, and qualitative variables are expressed as the absolute number and relative frequencies. Data normality was verified using the Shapiro-Wilk test, boxplots, histograms, and quantile comparison graphs. The evaluation of outcomes over time was verified using models of generalized estimation equations with the best-suited distribution, considering the dependence among the measures from the same patient. Results are presented as ratios or differences in means, 95% confidence intervals (95%CI), and p-values. For quality of life, score models with linear distribution were used, and for VAS, models with gamma distribution were used. Logistic models were employed to assess dimensions, and the probability of a patient not having any problems (health status 1) in relative to those with some or extreme problems (health status 2 or 3) was estimated. The results are presented as proportions, odds ratios, 95%CIs, and their respective p-values. Analyses were performed using SPSS software version 26.0 (IBM Corp., Armonk, NY, USA), and a significance level of 5% was adopted. Statistical analyses were performed by a biomedical statistician.

## RESULTS

### Sample characterization

A total of 212 patients were included in this study. The mean age of the participants was 54 (SD, 12) years; 79 patients (37.3%) were aged over 60 years and 151 (71.2%) were male. The median waiting period for transplantation was 210 days (quartile [Q] 1, 120 days; Q3, 334 days). The most frequent etiologies of liver cirrhosis were alcohol consumption (26.9%), viral hepatitis (22.2%), and cryptogenic cirrhosis (19.8%). Hepatocellular carcinoma was present in 66 patients (31.1%).

The mean body mass index was 27.2 (SD, 5.6) kg/m^2^; 27% of participants had a body mass index above 30.0 kg/m^2^. The median MELD score was 17 (Q1, 11; Q3, 24; maximum, 48). Half of the patients were discharged ten days after transplantation. The demographic and clinical characteristics of the patients are presented in table 1.

**Table 1:** Demographic and clinical patient characteristics

Characteristic	
Sex, male - n (%)	151 (71.2)
Age, years	54 (12)
Aged ≥60 years - n (%)	79 (37.3)
Length of hospital stay, days	10 (7-17)
Liver disease etiology - n (%)	
Alcohol	57 (26.9)
Autoimmune	16 (7.5)
Cryptogenic	42 (19.8)
Non-alcoholic steatohepatitis	13 (6.1)
Other	20 (9.4)
Familiar amyloid polyneuropathy	17 (8.0)
Viral	47 (22.2)
Hepatocellular carcinoma present	66 (31.1)
Body mass index, kg/m^2^ (n=211)	27.2 (5.6)
Body mass index ≥30 kg/m^2^ (n=211)	57 (27.0)
Time on waiting list, days (n=205)	210 (120-334)
MELD score (n=207)	17 (11-24)
Comorbidities and habits (n=211) - n (%)	
*Diabetes mellitus*	79 (37.4)
Hypertension	55 (26.1)
Smoking	54 (25.6)

Categorical variables are presented as absolute numbers (percentages). Continuous variables are presented as means (standard deviations) or medians (interquartile ranges).

MELD: Model for End-Stage Liver Disease.

Ten (4.7%) patients died during the study period, four within three months of transplantation, two in the following two months, and four more than six months after the procedure.

### Utility scores after transplantation

Improvements in all dimensions of the EQ-5D were observed over time. Table 2 shows the distribution of patient health status at the time of transplantation (T0) and at 3 (T3), 6 (T6), and 12 (T12) months after surgery, showing an increase in the percentage of patients reporting no problems.

**Table 2:** Changes in EQ-5D dimension scores over time

Dimension		T0	T3	T6	T12
Mobility					
	0	118 (60)	138 (85)	145 (90)	126 (89)
	1	77 (39)	23 (14)	16 (10)	15 (11)
	2	1 (1)	2 (1)	0 (0)	0 (0)
Self-care					
	0	139 (71)	155 (95)	152 (94)	138 (98)
	1	50 (26)	5 (3)	8 (5)	3 (2)
	2	7 (4)	3 (2)	1 (1)	0 (0)
Usual activities					
	0	87 (44)	130 (80)	142 (89)	131 (93)
	1	83 (42)	30 (18)	15 (9)	10 (7)
	2	26 (13)	3 (2)	3 (2)	0 (0)
Pain/discomfort					
	0	63 (34)	94 (67)	101 (76)	103 (77)
	1	100 (54)	44 (31)	29 (22)	30 (23)
	2	23 (12)	3 (2)	3 (2)	0 (0)
Anxiety/depression					
	0	62 (33)	104 (74)	110 (83)	100 (75)
	1	91 (49)	36 (26)	22 (17)	29 (22)
	2	33 (18)	0 (0)	1 (1)	4 (3)

Data are presented as absolute numbers (percentages). Response levels: 0, no problems; 1, some problems; and 2, extreme problems.

**Figure 1:**
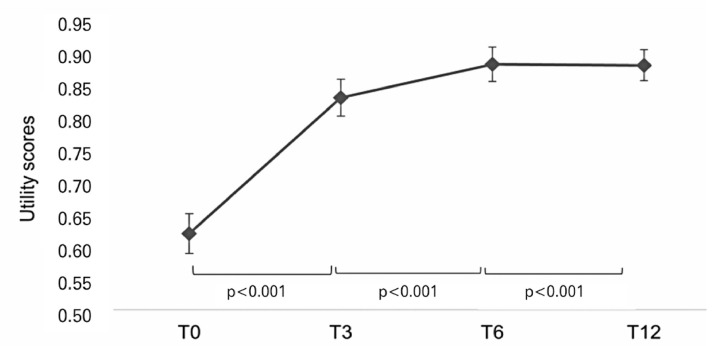
EQ-5D utility scores over time. Data are presented as estimated mean values and confidence intervals

Significant improvements in EQ-5D utility scores were observed at all post-transplantation time points compared with those obtained at T0, as shown in table 3 and figure 1. Compared with T0, the utility score increased by 0.22 points at T3 (p<0.001), 0.27 points at T6 (p<0.001), and 0.268 points at T12 (p<0.001). These data were reflected by the VAS results (Tables 1S and 2S and Figure 1S, Supplementary material).

**Table 3:** Changes in EQ-5D utility scores over time

Time point Minimum; Maximum	Mean (standard deviation)	Difference in means	p value
	95% CI		
T12 (n=131)			
	0.888 (0.148)	0.268	<0.001
	0.408 - 1	0.229; 0.308	
T6 (n=131)			
	0.892 (0.165)	0.270	<0.001
	0.305 - 1	0.229; 0.311	
T3 (n=137)			
	0.837 (0.177)	0.217	<0.001
	0.087 - 1	0.174; 0.26	
T0 (n=183)			
	0.622 (0.22)	Reference	-
	0.037 - 1		

95%CI: 95% confidence interval.

Compared with T3, there was an increase of 0.053 in the utility score at T6 (p=0.005) and 0.052 at T12 (p=0.008). No significant difference was observed between the utility scores at T12 and T6 (p=0.919).

### Comparison of changes in EQ-5D dimensions over time

In all domains of the EQ-5D, there was an increase in scores over time, demonstrating a significant improvement in patient HRQOL over time, with no significant difference between the periods evaluated (Table 4).

**Table 4:** Logistic models for EQ-5D dimension scores over time

Dimension		Proportion of patients with no problems	Odds ratios Inferior	95% CI		p value
				Superior		
Mobility						
	T12	0.90	5.655	3.057	10.46	<0.001
	T6	0.89	5.526	3.262	9.361	<0.001
	T3	0.86	3.953	2.328	6.712	<0.001
	T0	0.60	Reference	-	-	-
Self-care						
	T12	0.98	17.499	5.377	56.945	<0.001
	T6	0.94	6.099	3.154	11.793	<0.001
	T3	0.95	7.959	3.782	16.751	<0.001
	T0	0.72	Reference	-	-	-
Usual activities						
	T12	0.93	16.714	8.182	34.144	<0.001
	T6	0.89	9.646	5.619	16.558	<0.001
	T3	0.80	5.011	3.139	7.999	<0.001
	T0	0.44	Reference	-	-	-
Pain/discomfort						
	T12	0.78	6.730	4.087	11.083	< 0.001
	T6	0.76	6.059	3.648	10.062	< 0.001
	T3	0.67	3.852	2.377	6.242	< 0.001
	T0	0.34	Reference	-	-	-
Anxiety/depression						
	T12	0.75	6.165	3.866	9.832	<0.001
	T6	0.83	10.111	5.924	17.259	<0.001
	T3	0.75	5.955	3.771	9.403	<0.001
	T0	0.33	Reference	-	-	-

95%CI: 95% confidence interval.

## DISCUSSION

Although patient survival rates and clinical outcomes of LT have improved in recent decades, operative morbidity and the need for long-term patient care remain issues and may compromise HRQOL. However, in a large sample of patients, we observed a significant improvement in HRQOL at three months after LT, which was sustained during the one-year follow-up period.

A meta-analysis by Bravata et al. analyzed 49 studies involving 3,576 transplant recipients.^14^ Consistent with our findings, they reported that HRQOL was impaired during the pre-transplant period and improved after transplantation. Furthermore, significant gains were observed in the aspects of quality of life related to physical health, with smaller improvements in areas affected by psychological functioning.^14^ This trend toward a more significant improvement in physical quality of life was also suggested by a cohort study published in 2009.^15^ Conversely, using the EQ-5D, our data demonstrated significant improvements in both physical and psychological dimensions after transplantation.

Importantly, although a few studies have suggested that transplant recipients can achieve HRQOL levels similar to those of the general population, most have found that these patients retain a larger number of emotional and social issues.^16,17^ A cohort study used the Short-Form General Health Survey (SF-36) for quality of life to evaluate the HRQOL of 54 patients who underwent LT and 108 controls from the general population.^16^ Compared to the Control Group, LT recipients scored lower in most questionnaire domains, namely “mental composite score,” “physical activity,” “role limitations due to physical health,” “role limitations due to emotional state,” and “mental health.” The authors concluded by reinforcing the need for close monitoring of these patients and the provision of rehabilitation programs and regular psychosocial support.^16^ Studies in different populations have reported similar findings.^17^

Brazil is responsible for the second largest number of liver transplants performed annually; however, patients can experience long waiting periods with high MELD scores.^18,19^ Accordingly, our study population had a significant proportion of patients with high MELD scores who have a low quality of life due to liver disease symptoms and emotional and social distress.^20^ A Brazilian study performed between 2014 and 2016 used the Chronic Liver Disease Questionnaire to assess the HRQOL of 50 candidates for LT with chronic liver disease. Patients had an average quality of life, with a high proportion experiencing anxiety (52.0%) and depression (48.0%).^20^ The authors highlighted the need for healthcare professionals to consider HRQOL to help patients cope with the disease.

Our study showed a significant and progressive improvement in the HRQOL of liver transplant recipients after the procedure. Moreover, this improvement was observed as early as three months after LT in all dimensions evaluated by the questionnaire, and was sustained until the end of the one-year follow-up period. These data suggest the positive impact of LT on the HRQOL, even in patients with high MELD scores.

A Belgian study by Onghena et al. recently evaluated mental and physical HRQOL in 233 patients undergoing LT.^21^ The authors observed a decline in HRQOL in the first three months after surgery, a substantial improvement after one year, and a new setback two years after transplantation.^21^These findings differ from those of the present study, possibly because the mean MELD score (14.2 [SD, 6.8]) was lower than that of our population. This disparity may cause lower HRQOL scores in the pre-transplant assessment in our study, resulting in a significant improvement first three months after transplantation. Unfortunately, data regarding the impact of liver disease severity on the HRQOL of patients with cirrhosis waiting for transplantation are lacking.

The results of the current study suggest that LT can improve the HRQOL of patients with high MELD scores within a short time. This enhancement was sustained throughout the evaluation period, consistent with the findings of a systematic review of 33 studies involving 5,402 patients.^22^ However, other studies have reported contradictory results.^16,21^ The divergence in the literature probably reflects variabilities between recipient characteristics, regional and social aspects, and transplant centers. The significant improvements in HRQOL observed in the present study occurred despite the social and economic challenges inherent to developing countries, allied to the geographic limitations of their continental scale. We believe that the improvements observed are due to a structured and engaged multiprofessional care program that supports all aspects of patient life.

The present study has some limitations. First, this was a single-center retrospective study. Nevertheless, it involved a considerable number of patients and was performed at a high-volume tertiary transplant center with a structured multidisciplinary program. This novel study adds to the current body of evidence by systematically analyzing a large, prospectively maintained series of patients to investigate the impact of LT on HRQOL in a cohort of recipients with high MELD scores. However, prospective multicenter studies with predetermined follow-up time points are needed to define the predictors of increased HRQOL and implement development strategies, such as specific post-transplant multidisciplinary interventions, to further improve HRQOL.

## CONCLUSION

The significant improvement in health-related quality of life observed in transplant recipients with high MELD scores supports the use of complex surgical procedures in this population, although more data are needed to draw definitive conclusions. The observed improvements in health-related quality of life may be associated with the comprehensive multidisciplinary approach used in our center, which values all dimensions of patient health and health-related quality of life through a structured and individualized program. In addition, these results highlight the need for future studies to report liver disease severity before transplantation, as this factor affects the health-related quality of life of patients with cirrhosis.
