# Supplementary material for: Impact of liver transplantation on the quality of life of a cohort of high-risk recipients
Source: Einstein (Sao Paulo). 2025 Mar 28;23:eAO0565. doi: 10.31744/einstein_journal/2025AO0565 (PMC12014155; doi:10.31744/einstein_journal/2025AO0565)
Supplement: Supplementary file 1 [file 2317-6385-eins-23-eAO0565-suppl01.pdf]

SUPPLEMENTARY MATERIAL

Impact of liver transplantation on the quality of life of a cohort of high-risk recipients

Heloisa Barboza Paglione, Daisa de Mesquita Escobosa, Beatriz Mesquita Pimenta, Bianca Della-Guardia, Amanda Pinter Carvalheiro da Silva Boteon, Yuri Longatto Boteon

DOI: 10.31744/einstein_journal/2025AO0565

Table 1S. Changes in visual analog scale scores over time

Time point minimum; maximum	Mean (standard deviation) 95%CI	Difference in means	p value
T12 (n=133)			
	59.41 (20.22)	1.461	<0.001
	0 - 100	1.385; 1.541	
T6 (n=132)			
	84.64 (14.55)	1.465	<0.001
	9 - 100	1.389; 1.546	
T3 (n=138)			
	87.14 (12.67)	1.423	<0.001
	50 - 100	1.348; 1.502	
T0 (n=185)			
	86.71 (12.39)	Reference	-
	10 - 100		

95%CI: 95% confidence interval.

Table 2S. Estimated mean differences in visual analog scale scores

Time points compared	Difference in means (95%CI)	p value*
T3 -T0	25.157 (20.602; 29.713)	<0.001
T6 -T0	27.652 (23.006; 32.298)	<0.001
T12 -T0	27.409 (22.723; 32.096)	<0.001
T6 -T3	2.495 (-0.704; 5.694)	0.186
T12 -T3	2.252 (-1.166; 5.67)	0.279
T12 -T6	-0.243 (-3.072; 2.586)	0.866

*p value corrected using the sequential Bonferroni method.
95%CI: 95% confidence interval.

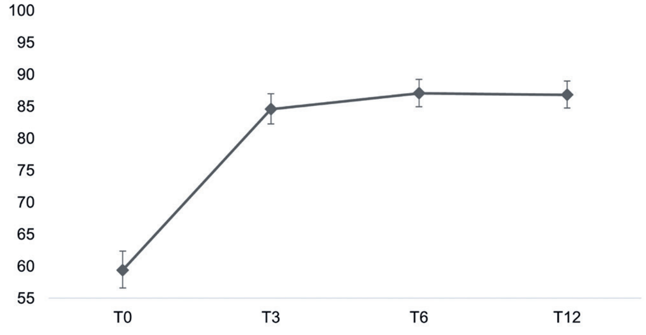

Figure 1S. Visual analog scale scores over time. Data are presented as estimated mean values and confidence intervals
